# Known but not called by name: recreational fishers’ ecological knowledge of freshwater plants in Hungary

**DOI:** 10.1186/s13002-021-00489-2

**Published:** 2021-11-04

**Authors:** Viktor Löki, Jenő Nagy, András Nagy, Dániel Babai, Zsolt Molnár, Balázs András Lukács

**Affiliations:** 1 Wetland Ecology Research Group, Institute of Aquatic Ecology, Centre for Ecological Research, Debrecen, Hungary; 2 Department of Evolutionary Zoology and Human Biology, University of Debrecen, Debrecen, Hungary; 3 Eötvös Loránd Research Network, Institute of Aquatic Ecology, Centre for Ecological Research, Budapest, Hungary; 4 Balatoni Road 62, Velence, Hungary; 5 Lendület Ethnoecology Research Group, Institute of Ethnology, Research Centre for the Humanities, Budapest, Hungary; 6 Traditional Ecological Knowledge Research Group, Institute of Ecology and Botany, Centre for Ecological Research, Vácrátót, Hungary

**Keywords:** Anglers, Ethnobiology, Ethnobotany, Conservation, Citizen science

## Abstract

**Background:**

Documenting local ecological knowledge (LEK) has recently become a topic of considerable interest. LEK can contribute to various areas of ecology, including habitat management and conservation biology. It has been recently revealed that recreational fishers’ ecological knowledge (FEK) can also provide valuable information about different organisms and habitats, while recreational fishers’ ecological knowledge is understudied in many aspects and regions of the world.

**Methods:**

We aimed to record Hungarian recreational FEK on plant species related to freshwater habitats. Our research was conducted in three regularly fished water bodies in Hungary, namely Lake Velence, Keleti Main Canal, and Lake Látóképi, where a total of 72 interviews were conducted with recreational anglers. During interviews, 24 plant species occurring at freshwater habitats with common or sporadic distribution were shown to anglers as single species or in congeneric pairs. Miscellaneous plant-related knowledge of anglers was also collected.

**Results:**

Anglers identified a total of 16 plant species. They used 45 botanical or folk names. An angler knew the name of 4.6 plants on average and recognized 7.4 other species without naming it. According to our detailed analysis, anglers were able to name or at least recognize those plant species which are somehow related to fishing activities, are salient, and/or common. Moreover, anglers at Lake Velence recognized less plant species; however, they also had less years of fishing experience compared to anglers of the other two locations.

**Conclusion:**

We found that recreational FEK exists even in the case of freshwater plants which are not the main focus of anglers. It is highly presumable that recreational fishers would be able to provide reliable ecologically related data for scientific research establishing future citizen science projects of nature conservation.

## Background

Local ecological knowledge (LEK) is a kind of intellectual property held by a specific group of people about their local ecosystems [1]. As well as LEK offering a great opportunity to involve different social groups to the assessment of nature by initiating a dialogue with them, evaluating LEK also represents a great approach to complex environmental problems by learning from locals who might be in the deepest connection with their surrounding environment [2]. LEK, therefore, can contribute to various areas of ecology, including habitat management [3] or conservation biology [4].

Freshwater habitats are listed among the most threatened ecosystems around the world, which are strongly affected by human-induced eutrophication, overexploitation, invasive species, pollution, and habitat degradation [5]. Human activities have a great impact on the ecological status of these ecosystems [6], as well as on their ecosystem services [7]. As fishers are one of the prime users of freshwater ecosystem services, evaluating fishers’ ecological knowledge (FEK) and introducing fishers as stakeholders in the decision-making process can be essential in designing effective conservation and management actions and preserving freshwater ecosystems throughout the world.

It has been proven that besides fish-related knowledge, as stock assessment [8], ecological decay [9], or different aspects of ethnozoology [10], FEK can be also effectively used in many other areas of both maritime and freshwater research, as fishers often take even involuntarily valuable observations of their environment. Therefore, they can also report reliable data about sources and indicators of marine pollution [11] or ecosystem modelling [12]. FEK on freshwater ecosystems also represents a great opportunity in filling gaps for monitoring biodiversity [13], or forming local guidance on habitat management [14, 15], while FEK can also be an effective tool in freshwater habitat restoration [16, 17]. Moreover, ecological knowledge of fishers can be efficiently used in the sustainable management of local freshwaters [18–20], while it has also been revealed that even recreational FEK can fill knowledge gaps in ecology [21]. These studies indicate that fishers and anglers can be interviewed successfully on many other ecological questions besides their best known topics, such as stock assessment, ethnoecology, or fish biology.

To solve complex ecological problems, freshwater biologists aim to collect data on both natural and anthropogenic factors that might have an impact on freshwater habitats. This includes also recreational fishing, which represents one of the most popular sports and hobbies all over the globe, as on average, 10% of the population of any country of the world is engaged in recreational fishing [22]. While the amount of research about LEK and FEK is constantly growing, studies about recreational anglers are still underrepresented worldwide: in a recent review on FEK [23], recreational fishers’ ecological knowledge has been represented by only two studies.

During their fishing activities, recreational anglers are connected to freshwater plants by many ways. They seek for traces of fish herbivory on plants, they choose fishing spots according to the surrounding vegetation and the density of aquatic plants, or they remove submerged plants species from their hooks. According to historical ethnographic publications of Hungary, fishers of the past centuries also knew and used many different freshwater plant species for different purposes [e.g. [24–26]. There are almost three-quarters of a million people (7.67% of the total Hungarian population) engaged in recreational fishing in the country, while their number still exponentially grows. Despite this fact, currently there is no study assessing recreational FEK in Hungary.

Ethnobiological researches most often examine the most obvious groups of living organisms resulting from the lifestyle of the community involved. For example, recreational or commercial fishers are mostly asked about fish [27, 28], or herders are mostly asked about forage plants and pasture vegetation [29, 30]. However, if we want to involve these stakeholder groups in the development of future habitat management plans, we believe it is also worth knowing how well they know other groups of organisms, even in the case of those organisms, which are at the periphery of their interests. In this study, we aimed to initiate a dialogue with Hungarian recreational anglers following this viewpoint, by investigating their knowledge about 24 different freshwater plant species which might appear around them during their fishing activities. We hypothesized that (i) anglers have reliable species knowledge at least among the most frequent plants; (ii) they have higher knowledge of those species which are somehow related to their fishing activities; and (iii) their knowledge is consistent among the different sampling sites.

## Methods

### Study areas

**Fig. 1:**
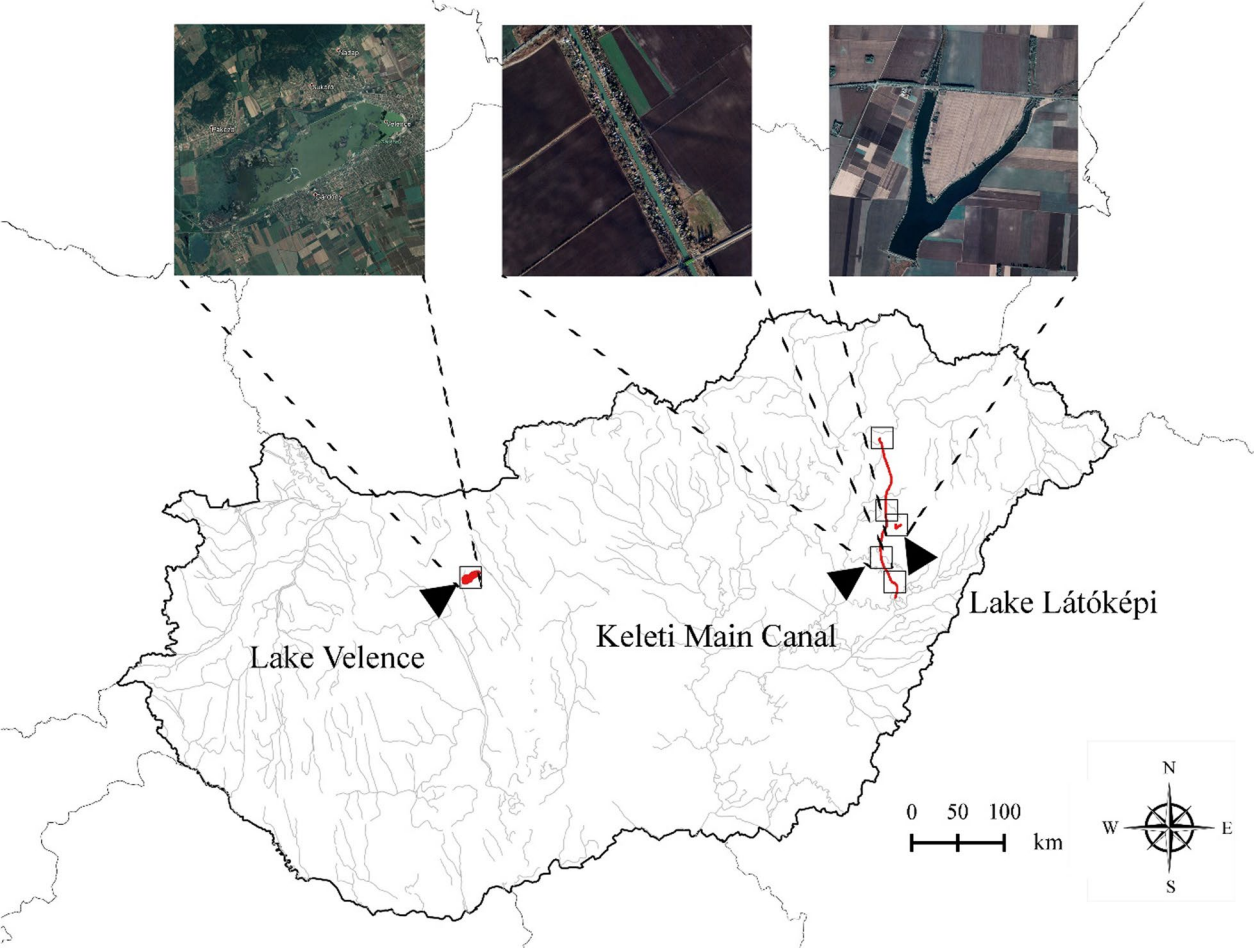
Map of the study areas in Hungary, Central Europe. Rectangles indicate localities of data collection. Country borders and water lines excluding the main rivers and lakes of the country (except for Lake Velence) are indicated with grey lines. Source of the base maps: Natural Earth; satellite images of study areas were exported from Google Earth Pro

Our research was conducted in three regularly fished water bodies of Hungary (Fig. 1), with significantly different vegetation. One of them represents a long-established artificial canal, while we also studied recreational FEK at a long-established reservoir, and a natural lake of the country.

The Keleti Main Canal is a 98 km long artificial watercourse between Tiszalök (Tisza) and Bakonszeg (Berettyó). The canal represents an important part of the sewerage system supporting water management in the region. The water transported in the canal is used to irrigate agricultural land, maintain fish farms, serve residential and industrial needs, or to reduce water shortages along the Körös River. Although it was initially planned, shipping has never materialized on the canal; while with its diverse waterfront vegetation, the canal also became important from a nature conservation point of view. The canal gives home to a total of 42 fish species [31].

Lake Látóképi is a 60 ha reservoir situated in the vicinity of Debrecen, the second largest city in Hungary. As a secondary function, it is managed as an important fishing water for anglers. As a total of 34 fish species are proven to live in the lake [32], this lake is the most visited fishing lake in the region with more than 9,000 purchased fishing tickets yearly. Due to the intensified management and intense activity of recreational anglers, the vegetation of the lake is strongly degraded, and extremely species poor.

Lake Velence is a large shallow lake in Hungary with an area of 26 km^2^. Because of the sunny climate and the shallowness of the lake, it is one of the warmest lakes in Europe, therefore, the number of aquatic plants with oligotrophic habitat requirements is limited here. The lake is also a popular holiday destination, while the south-western part of the lake is a nature conservation area maintaining diverse birdlife. A total of 28 fish species are known from the lake [33]. Among many freshwater habitats, floating reed islands can be found here with some valuable plant species.

### Data collection

Fieldwork was conducted between May and October 2020. To perform structured interviews [34], four different locations were visited in the whole length of the Keleti Main Canal, while anglers at one location were interviewed at Lake Látóképi, and also one at Lake Velence. Interviews were conducted in Hungarian. Before interviews, 24 plant species occurring at freshwater habitats with common or sporadic distribution were selected as single species or in congeneric pairs (see the list of plant species in Table 1), and were presented during interviews to the anglers by using a folder of coloured photographs. Every page showed one plant species on multiple images in full and in detail. Taxon names follow The Plant List [35]. The duration of the interviews varied between approximately 20 and 40 min. The initial interviewees preferred not to be taped, so in order to record the relevant information, extensive notes were taken during or immediately after the interview on pre-printed data sheets. The first part of the structured interview focused on the socio-demographic characteristics of anglers: age, gender, occupation, fishing experience, etc. (see detailed in Table 2). The second part was built to learn about their freshwater plant-related knowledge, by asking them a total of seven questions, but on the basis of one key question: (1) Do you know the name of the plant in these images? If an interviewee was unable identify a given plant by name, but claimed that he/she had already seen the plant in his/her life, to validate his/her knowledge about recognizing the species, we asked at least two of the following three questions to substantiate his/her claim: (2) When does the plant bloom? (3) What kind of habitat is preferred by this species? (4) Where exactly did you see it in the country? If a person could answer only one, or could not answer any of these three questions, his/her answer about recognizing the species was regarded as not confirmed. To learn more about the plants known by anglers, if an answer was confirmed, three additional questions were asked about the given species: (5) Do you know that this plant is legally protected, or not? (6) Do you know about the possibility of human consumption of this plant? (7) Do you know about any animals (including fish) which consume this plant?

**Table 1:** List of species in the questionnaire and the number of answers with relevant information

Scientific name	Relative frequency in the country (1: common; 5: rare)	Known by name (nr)	Known but not called by name (nr)	Unknown (nr)
*Phragmites australis* (Cav.) Trin. ex Steud.	1	70	2	–
*Nymphoides peltata* (S.G.Gmel.) Kuntze	3	1	31	40
*Nuphar lutea* (L.) Sm.	1	43	20	9
*Lemna trisulca* L.	2	–	14	58
*Lemna minor* L.	1	62	9	1
*Potamogeton nodosus* Poir.	2	–	41	31
*Stuckenia pectinata* (L.) Börner	2	–	30	42
*Hydrocharis morsus-ranae* L.	2	1	29	42
*Glyceria maxima* (Hartm.) Holmb.	1	–	38	34
*Sparganium emersum* Rehmann	3	1	11	60
*Sparganium erectum* Rehmann	1	1	11	60
*Trapa natans* L.	2	29	15	38
*Hippuris vulgaris* L.	5	–	7	65
*Ceratophyllum submersum* L.	3	1	6	65
*Ceratophyllum demersum* L.	2	1	62	9
*Juncus effusus* L.	1	–	35	37
*Juncus bufonius* L.	2	–	3	69
*Myriophyllum spicatum* L.	2	3	38	31
*Myriophyllum verticillatum* L.	3	3	36	33
*Iris pseudacorus* L.	1	33	28	11
*Nymphaea alba* L.	3	68	3	1
*Salvinia natans* (L.) All.	3	14	26	32
*Marsilea quadrifolia* L.	5	–	1	71
*Butomus umbellatus* L.	1	5	43	24

The relative frequency of the species was calculated based on the vascular plants of Hungary online database [76]. The order of species in the table follows the order of images in the questionnaire folder. The nomenclature follows The Plant List [35]

**Table 2:** Selected demographics of interviewed recreational fishers (*n* = 72)

	(%)	*n*
*Age*		
20–39	30	22
40–59	29	21
60 <	41	29
*Nature of occupation*		
Intellectual	28	20
Manual worker	48	34
Retired	24	18
*Fishing frequency*		
Every day	5	4
Weekly	63	45
Monthly	27	19
Yearly	5	4
*Fishing experience (years)*		
1–5	12	9
6–19	21	15
20–39	29	21
40 <	38	27
*Consuming caught fish*		
Yes	79	57
No	21	15
*Attending fishing competitions*		
Yes	22	16
No	78	56
*Fishing near to his residence*		
Yes	55	40
No	45	32
*Number of regularly visited fishing places*		
1	35	25
2–5	58	42
6–	7	6

Based on the answers for questions no. 1–4, we distinguished five categories: accurate answer (identified and named the plant by its official botanical name or by a documented folk name), likely accurate answer (named by a [local] name not [yet] documented in the Hungarian botanical, ethnographical, and ethnobiological literature), accurate identification (identified the plant [answered two of questions no. 2–4], but was unable to provide any name), likely inaccurate answer (unidentified the plant or reported a name which is likely the result of cognitive interference), and answer without information (i.e. ‘I do not know’, or no answer for the given question). For more details about used plant names, see Table 3.

**Table 3:** Names and cumulative number of identified plants by anglers

Scientific name	Hungarian name by angler(s)	Number of angler(s) reported the name	Confirmed dialectical name by mental representation (yes/no)	Confirmed botanical or folk name (yes/no)
*Phragmites australis* (Cav.) Trin. ex Steud.	nád	70	Yes	Yes
*Nymphoides peltata* (S.G.Gmel.) Kuntze	tündérfátyol	1	Yes	Yes
*Nuphar lutea* (L.) Sibth. & Sm.	tök	3	Yes	No
	töklevél	15	Yes	No
	tökvirág	1	Yes	No
	vízitök	22	Yes	Yes
	liliom	1	No	No
	vízililiom	1	No	No
*Lemna minor* L.	békalencse	53	Yes	Yes
	békanyál	4	Yes	No
	lencse	4	Yes	No
	vízilencse	1	Yes	No
*Hydrocharis morsus-ranae* L.	békatutaj	1	Yes	Yes
*Sparganium emersum* Rehmann*/erectum* L.	békabuzogány	1	Yes	Yes
*Trapa natans* L.	sulyom	23	Yes	Yes
	vízigesztenye	1	Yes	Yes
	kecskeköröm	1	Yes	Yes
	ördöghínár	1	Yes	No
	vízidió	1	Yes	Yes
*Ceratophyllum demersum/submersum* L.	tócsagaz	1	Yes	Yes
	balinhínár	1	Yes	Yes
*Myriophyllum spicatum/verticillatum* L.	süllőhínár	3	Yes	Yes
*Iris pseudacorus* L.	liliom	13	Yes	No
	vízililiom	6	Yes	No
	írisz	5	Yes	Yes
	víziírisz	1	Yes	Yes
	vízihagyma	1	No	No
	nőszirom	5	Yes	Yes
	mocsárinőszirom	1	Yes	Yes
	tavililiom	1	Yes	No
*Nymphaea alba* L.	liliom	1	Yes	No
	vízililiom	8	Yes	No
	tündérrózsa	12	Yes	Yes
	tavirózsa	43	Yes	Yes
	lótusz	1	Yes	No
*Salvinia natans* (L.) All.	rucaöröm	9	Yes	Yes
	rucarence	1	Yes	Yes
	folyófű	1	Yes	No
	vízipáfrány	1	Yes	No
	vízipajzsika	1	Yes	No
	panyola	1	Yes	Yes
	kákonya	1	Yes	Yes
*Butomus umbellatus* L.	vidrafű	1	No	No
	káka	4	Yes	Yes
	virágkáka	1	Yes	Yes

An answer for the name was considered valid if the person both identified and named the plant, or if the person presented a dialectical form of the valid official or folk name of the species after identification, but the mental representation on identifying the species was considered correct. An answer was considered likely inaccurate if neither the informant person’s dialectical name by his mental representation, or a valid official or folk name was uttered

### Recreational angler demographics

Of the 72 anglers interviewed; 71 were male and 1 was female. The youngest interviewed person was 21, while the oldest was 80 years old (mean: 51 years). The mean number of fishing experience of interviewed anglers was 28 years, while 27 of them (38%) had more than 40 years of fishing experience. The vast majority of anglers were blue-collar workers (*n* = 34, 48%), reported about weekly fishing frequency (*n* = 45, 63%), and regularly visits 2–5 fishing places (*n* = 42, 58%). For detailed demographics of the interviewed anglers, see Table 2.

### Statistical analyses

First, we assigned the plant species into categories according to their appearance (salient, non-salient), relatedness (fishing, non-fishing), and commonness (common, rare). All 24 species were categorized into four groups: (1) salient, fishing related and common (*n* = 4, group ID hereafter: ###), (2) salient, fishing related and rare (*n* = 13, ##X), (3) salient, non-fishing related and rare (*n* = 5, #XX), and (4) non-salient, non-fishing related and rare (*n* = 2, XXX). Then, we evaluated these categories by discriminant analysis [36] applied on the number of relevant information (Table 2). After calculating the total proportion of named or recognized species, we applied linear regression to estimate its association with the above described characteristics and categories.

Second, we calculated the proportion of plant species named or recognized by each angler and applied linear regression to investigate the relationships between the experience and fishing-related characteristics of anglers and their knowledge of freshwater plants. We performed a model selection procedure to average the estimated parameters in multiple models and to calculate variable importance values. Dunn’s post hoc test [37] was used to find the differences in plant recognition among the groups of categorical variables and to investigate the differences in other characteristics.

Finally, we used principal component analysis [38] to explore the contribution of each plant to the total variance in the recognition of the species. We calculated Cronbach’s alpha [39] and the reliability of the principal component analysis measured as θ (theta) [40]. Moreover, we were able to identify three groups of anglers applying k-means clustering [41] on the first six principal components, which groups correspond to the knowledge and experience of anglers on plant species.

All statistical analyses were performed in R v3.6.0 [42]. The ‘MuMIn’ package [43] for model selection, the ‘dunn.test’ package [44] for Dunn’s test, the ‘MASS’ package [45] for discriminant analysis, and the ‘Rcmdr’ package [46, 47] for Cronbach’s alpha were used for these specific calculations.

## Results

### Naming and recognizing plant species

During all interviews, anglers identified by using official botanical or folk names a total of 16 plant species from the questionnaire (for the list of species and the number of all answers with relevant information, see Table 2). An angler correctly named 1–10 plant species of the total 24 (mean: 4.6), recognized, but not called by name an addition of 3–14 species (mean: 7.4), while was unable to recognize a total of 4–20 plants (mean: 11.8). A total of 45 names were used by anglers (Table 3). Scientific or folk names of 7 plants emerged regularly (more than 10 total mentions), which includes *Phragmites australis* (70 identifications by the name of the plant), *Nymphaea alba* (68), *Lemna minor* (62), *Nuphar lutea* (43), *Iris pseudacorus* (33), *Trapa natans* (29), and *Salvinia natans* (14). In the case of morphologically highly similar congeneric species pairs, anglers did not differentiate *Sparganium emersum* and *Sparganium erectum*, *Myriophyllum spicatum* and *Myriophyllum verticillatum*, while the informants clearly distinguished *Ceratophyllum submersum* and *Ceratophyllum demersum*. Most anglers treated together, and did not distinguish the following floating or rooted aquatic plant species (commonly named ‘hínár’, ca. pondweed in Hungarian): *Potamogeton nodosus*, *Stuckenia pectinata*, *Trapa natans*, *Myriophyllum spicatum*, *Myriophyllum verticillatum*, and *Salvinia natans*, while *Ceratophyllum submersum* and *Ceratophyllum demersum* were also called ‘hínár’, but as mentioned above, was clearly distinguished by anglers.

During the interviews, we heard a total of 66 answers of 20 botanically dialectical names, but after understanding the mental representation of anglers by using the control questions of the questionnaire, and also considering the available Hungarian botanical, ethnographical, and ethnobiological literature, 62 (88.5%) of these answers related to 16 botanically dialectical name were considered as likely accurate, especially when the dialectical name was used consistently (see Table 3 for details). The names ‘lily’ (liliom) and ‘water lily’ (vízililiom) were used equally for *Nuphar lutea*, *Iris pseudacorus*, and *Nymphaea alba*; these are widely used names for both *I. pseudacorus* and *N. alba* in the country [48, 49]; therefore, the answers were accepted as dialectical names for the other two species. Five reported folk names, as ‘kecskeköröm’: (ca. goat-nail) for *Trapa natans*, ‘balinhínár’ (ca. asp’s pondweed) for *Myriophyllum spicatum/verticillatum*, ‘rucarence’ (ca. duck’s bladder-wort), ‘panyola’ and ‘kákonya’ (untranslatable names) for *Salvinia natans* need further investigations due to the limited number of reports, the origin, distribution, and other variants of these names (see all plant names and likely accurate answers also in Table 3).

**Fig. 2:**
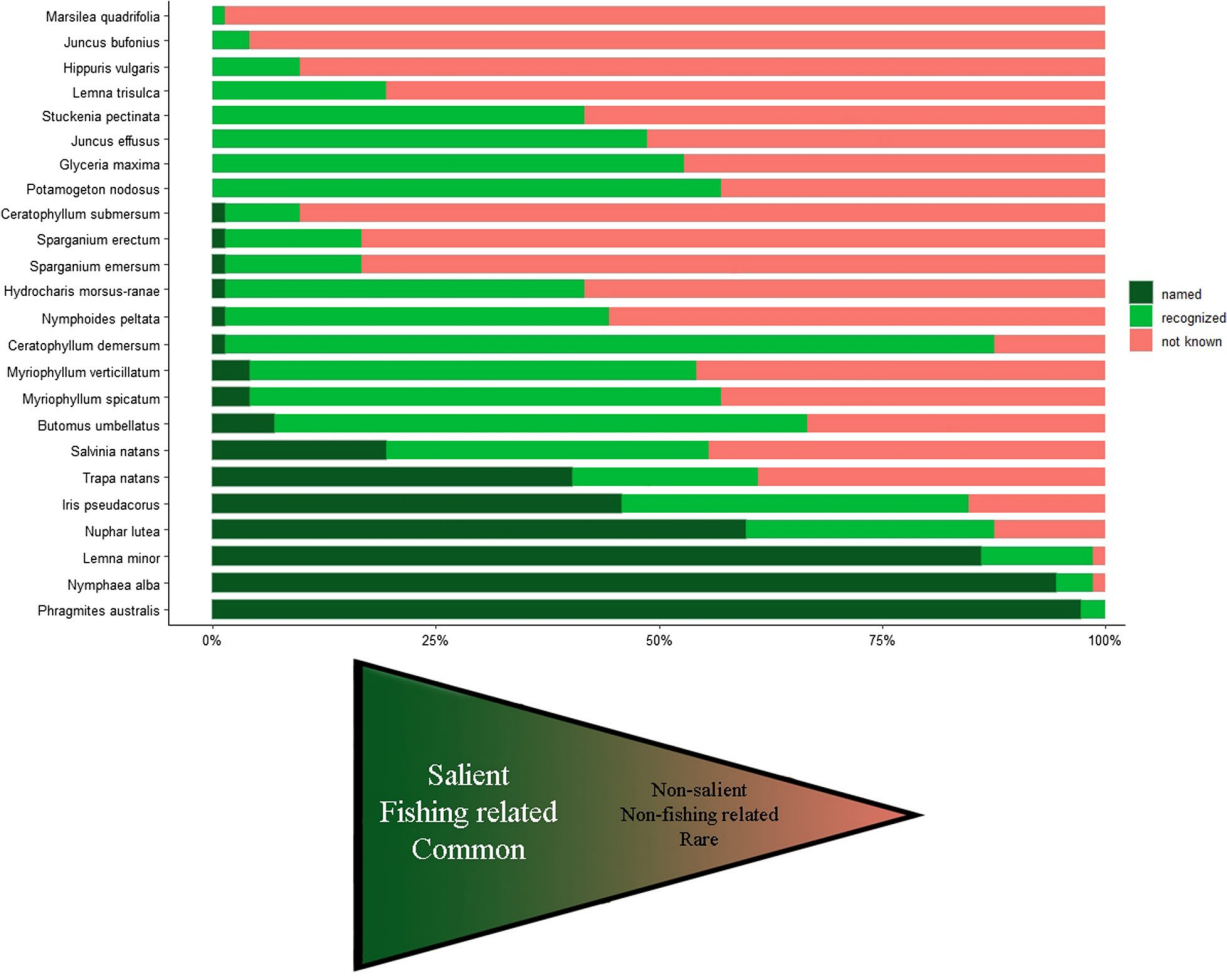
Proportion of answers with relevant information, and relative relatedness to anglers during their fishing activities. All plant species in the questionnaire were ordered by the following criteria: salient/non-salient, fishing related/non-fishing related, common/rare. For the total list of species and answers, see Table 1; for results of the discriminant analysis, see Figs. 4 and 5

**Fig. 3:**
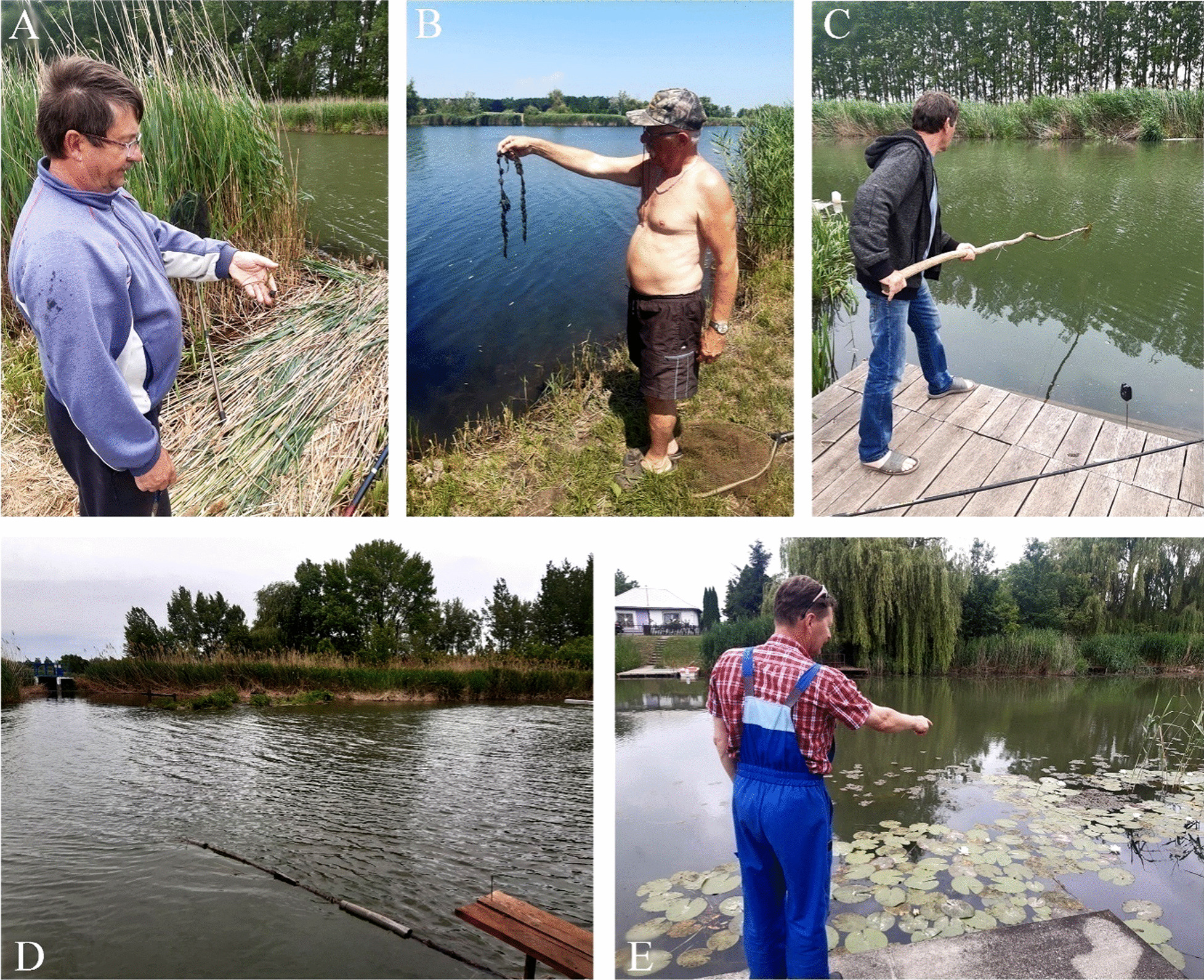
Key freshwater plants and some used plant-related tools of recreational fishers. **a** Germinating fruit of *Trapa natans* identified by an angler [Keleti Main Canal]; **b** well-known, but unnamed sprout of *Myriophyllum spicatum* removed from the hook [Lake Látóképi]; **c** River bed clearing with a long wooden stick by an angler to avoid hooking to plants anchored into the mud during fishing [Keleti Main Canal]; **d** an installed branch against floating aquatic plants in the front of a private fishing pier. The branch leads floating plants to the middle of the riverbed, thus keeps the water clear in the front of a fishing pier [Keleti Main Canal]; **e** an angler shows the spared population of the protected *Nymphaea alba* in the front of his fishing pier [Keleti Main Canal]. Photographs: **a**–**e** by Viktor Löki

Despite the fact that they could not name them, the interviewed anglers could recognize and had knowledge of many of the plant species, mostly which are salient, or somehow related to their fishing activities (Table 2; Figs. 2, 3). More than half of the interviewed anglers could answer to at least two of questions 2–4 (see Data collection in Methods) in the case of *Ceratophyllum demersum* (62 correct answers without the name of the plant), *Butomus umbellatus* (43), *Potamogeton nodosus* (41), *Glyceria maxima* (38), and *Myriophyllum spicatum* (38). At least half of the interviewed anglers did not know the name and were unable to recognize *Marsilea quadrifolia* (71), *Juncus bufonius* (69), *Hippuris vulgaris* (65), *Ceratophyllum submersum* (65), *Sparganium emersum* (60), *Sparganium demersum* (60), *Lemna trisulca* (58), *Stuckenia pectinata* (42), *Hydrocharis morsus-ranae (*42), *Nymphoides peltata* (40), and *Trapa natans* (38). None of the anglers could name *Lemna trisulca*, *Potamogeton nodosus*, *Stuckenia pectinata*, *Glyceria maxima*, *Hippuris vulgaris*, *Juncus effusus*, *Juncus bufonius*, and *Marsilea quadrifolia*.

### FEK of protected status and consumption by humans and wild animals

A total of 38 anglers (52%) reported that *Nymphaea alba* is legally protected. Only six of the anglers (8%) mentioned the protected status of *Trapa natans*, while no other legally protected plant species in the questionnaire (*Nymphoides peltata*, *Hippuris vulgaris*, *Salvinia natans*, *Marsilea quadrifolia*) were mentioned during the interviews. To our question about the possible human consumption, six anglers (8%) reported that *Trapa natans* is edible, while no other plants were mentioned for possible human consumption. For the question ‘Do you know about any animals (including fish) which consume this plant?’, 48 anglers (66%) reported that *Ctenopharyngodon idella* regularly consumes fresh or mature sprouts of *Phragmites australis*, while other relevant plant consuming fish, bird, or mammal species were reported scarcely (bird species—19 times; other fish species—18 times; mammal species—8 times, mainly reporting that animals occurring around freshwater habitats consuming ‘pondweeds’ in general), and except for reporting the *Ctenopharyngodon idella*, half of the anglers (*n* = 36) did not mention any animals which consume any of the plant species specifically.

### Anglers miscellaneous perceptions on freshwater plant species and their surroundings

While anglers formed neutral opinions of most of the floating or rooted plant species, some of the species repeatedly suffered from rather negative judgements: Trapa natans—‘long branches are needed in the front of the fishing piers against them (see Fig. 3)’; ‘if I can reach from my pier, I always pull out the rosettes from the water’; ‘it is an aggressive plant, it can even germinate from 2 m under water’; ‘this plant is not legally protected for sure, or if yes, whoever protects it is not normal’, Ceratophyllum demersum—‘it always gets stuck on the hook, I hate it’; ‘it destroys the boat’s engine, and squeezing the clutch’, Salvinia natans—‘if it multiplies, you can collect it all day long’, aquatic plants in general—‘the fish taste of mud mainly because of this plant, it needs to be exterminated’; ‘it needs to be exterminated, it becomes entangled in the fishing line’.

During the interviews, regardless of the questions asked, some of the anglers reported information about the current status of vegetation (e.g. ‘There are too dense patches of *Phragmites australis* here, it has to be cut at the winter’), some of them observed and verbalized long-time changes in the vegetation and density of selected plant species (e.g. ‘*Trapa natans* was less widespread 30 years ago then now’) or in habitat management (e.g. ‘The canal is very turbid, so dredging is urgently needed here’).

While only one of the interviewees knew the name of *Nymphoides peltata*, 31 other anglers (43% of the total interviewees) had seen the plant before, and 23 anglers (31% of the total interviewees) reported about specific sites of the species in the country, mostly Lake Tisza, which represents the greatest population of the plant in the country. Seven interviewees even at Lake Velence remembered this species from Lake Tisza. Three anglers reported negative experiences about struggling by boat in a dense population of *N. peltata* (e.g. ‘There is so much of this plant in the Lake Tisza that you can barely move in the plant mass with the boat’).

### Factors affecting FEK

**Fig. 4:**
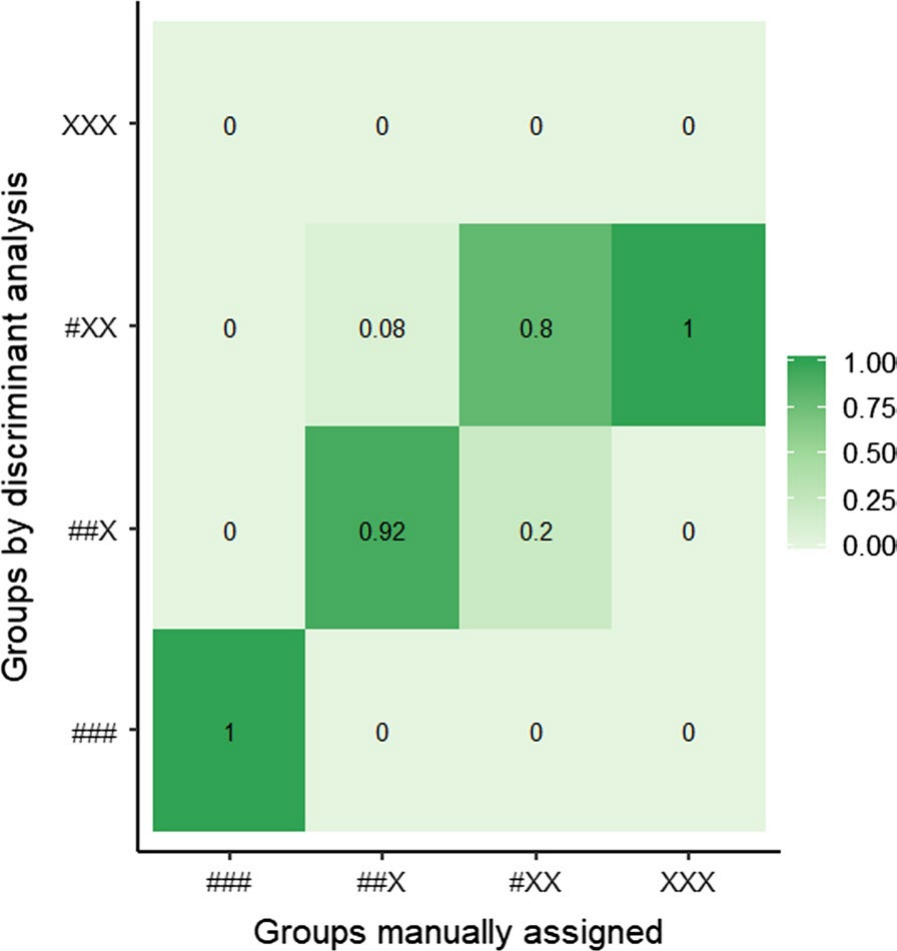
The proportion of identically defined groups comparing our manual categorization with the results of the discriminant analysis. The values in the diagonal indicate the correctly assigned groups (###: salient, fishing related and common, ##X: salient, fishing related and rare, #XX: salient, non-fishing related and rare, XXX: non-salient, non-fishing related and rare)

Our categorization of plants, including characteristics of appearance, relatedness and commonness, was confirmed by the results of discriminant analysis. The average proportion of correctly identified categories was 0.83 (range 0.8–1, Fig. 4) and only the two species in the fourth category (non-salient, non-fishing related, rare) were assigned to the third category (salient, non-fishing related, rare). The proportion of named or recognized species is significantly lower in categories with decreasing relatedness to fishing and commonness (*R*^2^ = 0.74, ### (intercept), *β* = 0.96, *t* = 11.23, *P* < 0.001; ##X, *β* = −0.41, *t* = −4.2, *P* < 0.001; #XX, *β* = −0.75, *t* = −6.53, *P* < 0.001; XXX, *β* = −0.91, *t* = −6.11, *P* < 0.001). However, by estimating the relationships between the same proportion and each of the characteristics (*R*^2^ = 0.74), we found significant associations in relatedness and commonness (*β* = 0.34, *t* = 3.75, *P* = 0.001 and *β* = 0.41, *t* = 4.21, *P* < 0.001, respectively), but not in salience (*β* = 0.16, *t* = 1.09, *P* = 0.29). According to the results of our linear models, some of the components of fishing experience, namely the years spent with fishing (*β* = 0.003, *t* = 2.55, *P* = 0.013) and the number of visited locations (*β* = 0.01, *t* = 3.07, *P* = 0.003) showed significant differences in the proportion of named or recognized plant species. Furthermore, only Lake Velence had significant association with species number (*β* = −0.1, *t* = −2.23, *P* = 0.029), where anglers recognized less plant species compared to Keleti Main Canal. These results were also confirmed by the model selection (Tables 4, 5).

**Table 4:** Comparison of truly competitive models of the proportion of plant recognition and its predictors in the best subset (ΔAICc < 4) of models, ordered by AICc values

Model No.	Predictors	Df	logLik	AICc	ΔAICc	Weight
1	Number of fishing locations + years of fishing	4	52.51	− 96.43	0.14	0.29
2	Age + number of fishing locations + years of fishing	5	52.97	− 95.03	1.55	0.14
3	Number of fishing locations + site	5	52.44	− 93.98	2.60	0.09
4	Number of fishing locations + job type + site + years of fishing	8	55.90	− 93.52	3.05	0.07
5	Number of fishing locations + residence + site	6	53.30	− 93.30	3.27	0.06
6	Age + number of fishing locations + residence + site + years of fishing	8	55.76	− 93.23	3.34	0.06
7	Frequency of fishing + number of fishing locations + years of fishing	7	54.41	− 93.07	3.50	0.05
8	Years at the current location + number of fishing locations + site	6	53.14	− 92.98	3.59	0.05
9	Age + fishing competition + number of fishing locations + years of fishing	6	53.06	− 92.83	3.74	0.05
10	Age + years at the current location + number of fishing locations + years of fishing	6	53.03	− 92.76	3.81	0.05
11	Age + fishing for consumption + number of fishing locations + years of fishing	6	53.02	− 92.75	3.82	0.05
12	Age + number of fishing locations + residence + years of fishing	6	52.99	− 92.68	3.89	0.04

**Table 5:** Model-averaged parameter estimates (*β*), standard errors (SE), and variable importance (*I*) for the predictors of plant recognition

Predictor	*β*	SE	*I*
Number of fishing locations	0.014	0.004	1
Years of fishing	0.003	0.001	0.8
Age	− 0.001	0.001	0.39
Site (Keleti)			0.32
Site (Látóképi)	− 0.045	0.039	
Site (Velence)	− 0.099	0.041	
Job type (blue-collar)			0.07
Job type (retired)	− 0.016	0.039	
Job type (white-collar)	0.036	0.033	
Residence	0.025	0.037	0.16
Fishing frequency (daily)			0.05
Fishing frequency (monthly)	0.049	0.067	
Fishing frequency (weekly)	0.087	0.062	
Fishing frequency (yearly)	0.023	0.087	
Years at the current location	0.001	0.001	0.1
Fishing competition	0.015	0.035	0.05
Fishing for consumption	0.011	0.036	0.05

Levels of categorical variables shown in parentheses were averaged separately

The fact that anglers better recognized aquatic plant species alongside Keleti Main Canal compared to Lake Velence was also confirmed by Dunn’s test (*Z* = 2.7, *P* = 0.02). Any other comparisons for the categorical predictors were statistically non-significant; however, we were interested in the differences in other fishing-related experiences of anglers among fishing sites, among job types and in the frequency of fishing. We found that retired anglers are fishing significantly longer than people in blue-collar (*Z* = 3.2, *P* = 0.02) or white-collar jobs (*Z* = 3.2, *P* = 0.04) and similarly, anglers at Lake Látóképi or at Keleti Main Canal have started fishing earlier in their life compared to Lake Velence (*Z* = 2.81, *P* = 0.007; *Z* = 3.09, *P* = 0.006, respectively). There were no significant differences in the rest of the comparisons.

**Fig. 5:**
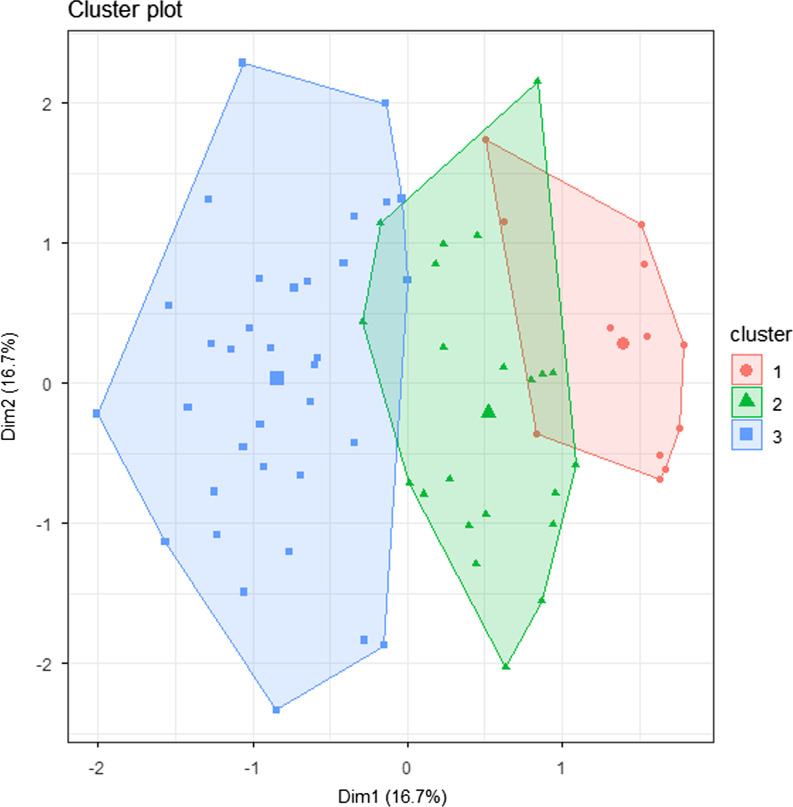
Cluster groups by *k*-means clustering based on six principal components of plant recognition data as input

The 24 plant species represent different levels of their recognition, therefore, they contributed unequally to the knowledge of anglers. We applied principal component analysis on the raw values (named, recognized, and not known) given for each species based on the answers of the 72 interviewees. The average of Cronbach’s alpha was 0.729 and the θ was 0.77 when we included all species in the analysis. However, excluding some of the variables resulted in higher reliability of the analysis. After removing 11 items out of 24, Cronbach’s alpha became 0.777 and θ was 0.79. We extracted six principal components (PC1 to PC6), explaining 83.43% of the total variance, for further analysis. PC1 represented the recognition of *T. natans* and *S. natans*, PC2 was highly contributed by *Iris pseudacorus*, *Nuphar lutea*, and *T. natans*, while PC3 was characterized by *Sparganium emersum*, *S. erectum*, and *I. pseudacorus* (see more details in Table 6). Using the six principal components for k-mean clustering, we identified 3 groups (Fig. 5). Anglers in group 1 are fishing significantly shorter than in groups 2 or 3 (Dunn’s test, group 1–group 2: *Z* = −2.27, *P* = 0.03; group 1–group 3: *Z* = −3.67, *P* < 0.001), are visiting the current fishing location more recently than people in group 3 (*Z* = −3.13, *P* = 0.005). The proportion of named or recognized plant species was decreasing among the groups (group 1–group 2: *Z* = −2.45, *P* = 0.01; group 1–group 3: *Z* = −5.42, *P* < 0.001; group 2–group 3: *Z* = −3.39, *P* = 0.001). Groups were not statistically different in other comparisons, as well as, we did not find significant differences in age and the number of fishing locations regularly visited among the groups.

**Table 6:** Variable loadings on principal components

	PC1	PC2	PC3	PC4	PC5	PC6
*Nymphoides peltata*	− 0.111	0.1356	0.169	− 0.0579	− 0.6289	− 0.1032
*Nuphar lutea*	− 0.3058	0.4991	− 0.2301	0.6623	− 0.0283	− 0.0354
*Lemna minor*	− 0.0818	0.161	0.0095	0.2666	0.0416	− 0.1168
*Potamogeton nodosus*	− 0.2145	− 0.0345	0.063	− 0.2067	− 0.3276	0.2175
*Hydrocharis morsus-ranae*	− 0.2396	− 0.2134	− 0.2546	− 0.1065	− 0.0734	0.5461
*Sparganium emersum*	− 0.0794	0.0776	− 0.5234	− 0.2998	0.0249	− 0.3356
*Sparganium erectum*	− 0.0794	0.0776	− 0.5234	− 0.2998	0.0249	− 0.3356
*Trapa natans*	− 0.603	− 0.4113	0.2817	0.0578	− 0.0973	− 0.5019
*Ceratophyllum demersum*	− 0.0848	0.1334	− 0.0439	0.008	0.1079	0.1239
*Juncus bufonius*	− 0.0287	0.0257	− 0.0341	− 0.0709	0.0184	− 0.0197
*Iris pseudacorus*	− 0.2817	0.551	0.4292	− 0.4538	0.4063	− 0.0016
*Salvinia natans*	− 0.4943	− 0.2702	− 0.1462	0.0863	0.3995	0.2605
*Butomus umbellatus*	− 0.2759	0.2891	− 0.1174	− 0.1767	− 0.3766	0.261

Negative numbers represent lower values in the raw data, thus, they indicate that anglers are more likely to name or recognize the species

## Discussion

### Depth and certainty of recreational anglers’ plant knowledge

In this study, we aimed to open a dialogue to learn more about recreational FEK on a selected list of freshwater plants. The study revealed that anglers were able to provide valuable data about freshwater plant species, which were not in their main focus during fishing activities; therefore, we suggest that recreational anglers could be effectively involved in future thematic citizen science projects related even to freshwater plants.

During the interviews, anglers mentioned official botanical or folk names for 16 of the 24 species in the questionnaire. An angler correctly named an average of 4.6 plants and additionally recognized but was not able to name an average of 7.4 plants. This means that an interviewed angler was able to recognize and talk about, on average, half of the shown plant species. Anglers’ plant knowledge was limited even of some common species, and many species were not named, which may suggest limited knowledge, and/or knowledge fragmentation, especially compared to the large number of plant names used by traditional shepherds of the Hortobágy region [50]. This is also confirmed by the fact that the number of reported folk names (7) was low (15.5% of all mentioned names). However, surprisingly, five reported folk names require further interviews. This may have occurred due to there being many Hungarian landscapes that are not yet covered by systematic linguistic or ethnobiological folk name research, while it is highly presumable that knowledge erosion is also high, so rare, hitherto unknown names may occur during such researches.

According to our expectations, anglers were more likely to identify the most common and fishing-related plant species, while the proportion of named or known species significantly decreased by reaching plant species which are rare or marginally related to fishing activities. More specifically, the most significant components of species recognition were plant frequency and presence during the fishing activity; however, the appearance of plant species alone had no significant effect on the proportion of named or known species (see Figs. 2, 4). We found a relatively high number (10, 41.6%) of plants that were well known to anglers, but with a few exceptions, they did not call them by their names. It seems that many of the plant species were salient enough for anglers to recognize, but if a species was outside of their fishing interests, they mostly did not call them by names. Naming a plant requires the operation of some pathway of knowledge transmission [51]: this is how the given name can be passed on, which is—according to these results—does not seem to work in the community of Hungarian anglers. In parallel with this, it is need to be mentioned that other knowledge of various species, habitats, or population dynamics is probably the result of personal experience of anglers.

Neither of the non-salient nor the rare plants were named in the present study: this is also not surprising when considering that generally freshwater plants were not in the centre of anglers' interests during fishing. Therefore, we suggest that one of the main results of the present study is that anglers were able to talk about those freshwater plants which were salient, or somehow related to their fishing activities. This can also be an indirect evidence that the best of their knowledge was not likely acquired in school. This conclusion is further strengthened by two facts: (1) seven interviewees even at Lake Velence remembered *Nymphoides peltata* from Lake Tisza. The accurate observations of many anglers independent of each other can presumably be mainly due to the perceptive and morphological salience of the plant, while cultural salience also contributed to memorizing the species. (2) Similarly to other interviewed social groups [52], anglers’ knowledge on the protected status of plants was severely incomplete, except for *Nymphaea alba*, as more than half of the interviewed anglers (52%) reported the protected status of this plant. This can be associated with the significant morphological salience of this species on Hungarian fishing waters, while cultural salience may also contribute for this: an excellent example for the contribution of cultural salience was the interviewed angler at the upper section of Keleti Main Canal, who correctly identified *Nymphaea alba,* knew both the name and the habitat preference of the plant, but after confessed that he never saw it in the nature, told us that he knows the plant from the Hungarian animated film ‘Szaffi’. The latter phenomenon is not surprising in the light of the fact that people often learn aquatic plants early, as some of them often have striking form so that they even occur in illustrations for children books: for example, *Typha* spp., *Iris* spp., and *Nymphaea alba* were one of the most frequent plants occurring in such illustrations, both in Poland and Britain [53].

Interviewed anglers could not make any difference in any of the congeneric species pairs of the questionnaire with the exception of *Ceratophyllum demersum* and *C. submersum*. These two plants were strongly related to anglers' fishing activities in this region. *Ceratophyllum* species are often stuck on their hooks, thus they could regularly observe these two species up close. Although anglers were not distinguishing these two species with separate names, due to the frequent physical contact with them, they still could recognize the slight morphological differences, of which the main difference between the two plants is the rough sense to the touch in *C. demersum*, and soft in *C. submersum*. However, among traditional people all around the world, the folk generic level is the level of general knowledge, and species within any genus are rarely distinguished [54]. Besides this, most anglers treated together with the vast majority of floating or rooted macrophytes and named them ‘hínár’ (ca. pondweed). This is also a general phenomenon in the case of shepherds of Hortobágy: many unused or irrelevant species for them is treated under a collective name, while only some of them have a separate name [50, 55].

### The role of experience on field and fishing location in anglers’ knowledge

The general experience is that ecological knowledge increases with age [56, 57]. This can be also observed in the case of fishers: Ainsworth & Pitcher [58] after evaluating LEK of commercial, aboriginal, and recreational fishers found that interviewees with 40 or more years of experience provide a significantly higher fraction of comments that agree with stock assessment than less experienced ones. During our study, considering that anglers have different years of experience in fishing and also the recognition of the 24 plant species of the questionnaire requires a different level of knowledge, we attempted to identify groups among anglers based on their answers during the interviews. According to the results of the cluster analysis, anglers have a shared preference in fishing activities, specifically, they visit the current fishing location more frequently but have started to fish more recently in one of the three groups identified. The level of knowledge on the studied plant species is decreasing by groups (1, 2, and 3, respectively), which can be explained by the expertise in fishing and the visited locations. Groups 2 and 3 included anglers with an increased level of fishing experience and they more often visited Lake Látóképi and Keleti Main Canal, respectively. As elder anglers are likely able to provide more ecologically valuable information, we suggest that they could also possibly report about long-term dynamics or changes of freshwater habitats, as it has already been proven that in certain cases only experienced fishers can provide historical data at many waters of the world [18]. In parallel with this, as Davis and Wagner [59] suggested, in order to learn more about nature, it can be also important to identify and to select ‘local knowledge experts’.

We found that anglers visiting various fishing locations and/or having more years of fishing experience knew and named a larger proportion of plant species. However, it seems that the fishing location (more specifically: the angler community of a given fishing water) can also affect the number of recognized plant species since anglers were generally less familiar with plant species at Lake Velence compared to anglers of the other two locations. Although detailed knowledge and possible knowledge gaps of different fishing regions need further studies, this fact can be partially explained by at least three reasons: (1) Lake Velence is mainly visited by anglers from the capital of Hungary (Budapest); (2) anglers at the Lake Velence had less years of fishing experience; (3) anglers at Lake Látóképi or Keleti Main Canal have started fishing earlier in their life compared to Lake Velence. Based on these results, it seems that ecological knowledge of recreational anglers about freshwater plants is also significantly influenced by the years spent in the field, as if shared information is limited, personal experience and observation is the most important source of TEK [60, 61]. Although this is yet to be investigated concerning other organisms and ecologically related information, in our view, it can determine an important direction for approaching recreational FEK in future studies.

### Miscellaneous observations on-field by anglers

During the interviews, anglers also had proven that they can provide valuable data as miscellaneous ecological information of these selected species. However, except for reporting that *Ctenopharyngodon idella* regularly consumes fresh or mature sprouts of *Phragmites australis*, other relevant plant consuming fish, bird, or mammal species were reported scarcely during the interviews. This might be also an indirect evidence of knowledge fragmentation among recreational anglers, as plants that were widely used or eaten (e.g. *Phragmites australis*, *Trapa natans*, *Lemna minor*, *Butomus umbellatus*, etc*.*) for various reasons in the past were still known by name in some cases. It is highly presumable that as very few anglers reported about any possible uses of plants, due to the radical changes in the lifestyle of previous generations, traditional uses of these plants were mostly immersed in obscurity for recreational anglers in the past few decades. This also can be a piece of indirect evidence for the lack of their interest in the vast majority of plants: their traditional uses have been mostly forgotten, they do not consume these plants anymore, and it seems that with a few exceptions, they do not link them to various aspects of fish biology and behaviour. In a different view, however, considering their low level of general interest in freshwater plants, their involuntary observations on many freshwater plant species also praise the recreational anglers' sharp eyes out in nature.

### Potentials for collaboration of anglers, scientists, and freshwater managers

The evaluation of ecosystems for different purposes by professionals mostly represents time-consuming and expensive activities, which also mostly requires extensive funding [4]. By organizing citizen science projects, many groups of the modern society can be involved effectively in scientific data collection. It has recently been proven that citizen science can significantly improve natural resource management, environmental protection, and even conservation science [62]. Combining scientific methods applied by professionals and relevant ecological knowledge of different stakeholder groups has opened new possibilities to learn more about nature, while this kind of knowledge co-production luckily, can also build partnership and community consensus between scientists and the involved social groups [63]. In other words: bridging the methods of professional conservationists together with LEK and implementing co-production of knowledge can be essential in designing the best possible habitat management practices in the future [64, 65]. We believe that these are also true in the case of recreational FEK, and that dialogue will hopefully improve in the coming years, as based on their knowledge, anglers could be involved in different participatory monitoring actions.

In this study, we were able to present that anglers can form definite opinions about certain (fishing related) plant species, different freshwater habitats, or some observed long-time changes of fishing waters. It is highly presumable that by applying additional thematic interviews in the future, anglers would be able to provide valuable ecologically related data in different aspects of freshwater habitats; although it is need to be mentioned that while indicators of folk landscape change are often similar, sometimes they can be different from those used by science [66].

While anglers’ ecological knowledge possibly could be used in the future for solving many freshwater-related problems, integrating fishers’ activities [67, 68] and knowledge in habitat management is definitely still a challenge [69, 70], while engaging recreational fishers in the management and conservation issues of freshwaters is an important, yet mostly unresolved problem [71]. As plants are also generally not in the centre of anglers’ main interest, we suggest that to maximize the data collecting efficiency about plants, the possible thematic data collection mechanism in the future should also follow this decentralized approach. It has been proven that social media [72] or using an online video sharing platform [73] can provide complementary data for recreational fisheries research; therefore, a great opportunity for collecting data on selected plant species from anglers could be the use of mobile applications for recreational fishers, where they voluntarily report about their catch and some other circumstances of catch (fishing site, weather conditions, fishing methods, etc.). Several such applications are available and being developed worldwide (e.g. the international *FishAngler*, with more than 500.000 downloads [74], or the Hungarian *Fishinda* with more than 100.000 downloads [75] by the date 25/05/2021), and by integrating some relevant and well-tested questions in these applications about freshwater plants (or after testing, some fishing-related animals) would provide an extremely low-cost, but reliable, and still very information-rich data source. Due to a large number of anglers and the relatively good distribution of fishing bodies, even a database with national coverage could be built in this way.

As the traditional fishing lifestyle is disappearing throughout the world, the opportunity to learn more about their rich knowledge is decreasing from year to year, and in these circumstances, interviewing recreational anglers offers a great alternative opportunity to provide various types of valuable data about marine ecosystems, or as shown in the present study, about freshwaters. Other interviews, or as mentioned above, such digital applications offer a great opportunity to collect a high amount of quality data with the contribution of recreational anglers about freshwaters, including water quality, habitat management, their observations of selected freshwater-related animal or plant species, their opinion on the effects of ground baiting on water quality, etc., which opportunity is yet to be exploited in the future.

## Conclusions

While it has been recently revealed that recreational fishers’ ecological knowledge (recreational FEK) can provide valuable information about different organisms and habitats, recreational FEK is understudied in many aspects and regions of the world. We present the first study aiming to record Hungarian recreational FEK on 24 plant species related to freshwater habitats. As interviewed anglers identified a total of 16 plant species, and they used 45 botanical or folk names during interviews, we suggest that recreational FEK exists even in the case of freshwater plants which are not the main focus of anglers. Moreover, our detailed analysis showed that anglers were able to name or at least recognize those plant species which are somehow related to fishing activities, are salient, and/or common. According to the present study, it is highly presumable that recreational fishers would be able to provide reliable ecologically related data for scientific research establishing future citizen science projects of nature conservation.

## Data Availability

The datasets used and/or analysed during the current study are available from the corresponding author on reasonable request.
